# Osseointegration of Dental Implants after Vacuum Plasma Surface Treatment In Vivo

**DOI:** 10.3390/jfb15100278

**Published:** 2024-09-24

**Authors:** Se Hoon Kahm, Sang Hwa Lee, Youbong Lim, Hyun Jeong Jeon, Kyoung-In Yun

**Affiliations:** 1 Department of Dentistry, Eunpyeong St. Mary’s Hospital, College of Medicine, The Catholic University of Korea, 1021, Tongil-ro, Eunpyeong-gu, Seoul 03312, Republic of Korea; implant@catholic.ac.kr (S.H.K.); justina@catholic.ac.kr (S.H.L.); 2 Plasmapp Co., Ltd., 9, Giheungdanji-ro 24beon-gil, Giheung-gu, Yongin-si 17086, Republic of Korea; ceo@plasmapp.com (Y.L.); hjjeon@plasmapp.com (H.J.J.); 3 Department of Dentistry (Oral and Maxillofacial Surgery), Yeouido St. Mary’s Hospital, College of Medicine, The Catholic University of Korea, 10, 63-ro, Yeongdeungpo-gu, Seoul 07345, Republic of Korea

**Keywords:** dental implants, osseointegration, bone regeneration, biomedical and dental materials, implant surface ageing

## Abstract

Previous studies have highlighted the need for post-treatment of implants due to surface aging. This study investigated the effect of vacuum plasma (VP) treatment on the osseointegration of sandblasted, large grit, acid-etched (SLA) implant surfaces. The hypothesis was that VP might enhance implant stability, measured by implant stability quotient (ISQ) and histological osseointegration through bone-to-implant contact (BIC) and bone area ratio (BA) in rabbit models. Eighteen implants were either untreated or treated with VP and installed into the femurs of six rabbits, which were sacrificed after four weeks. Histological analyses of BIC and BA, along with micro-CT analysis of bone volume and ISQ, were performed. The VP-treated group showed higher levels of BA, bone volume, and ISQ, but no statistically significant differences were observed between the control and experimental groups. Despite limitations, both groups achieved better osseointegration and regeneration, warranting further studies on plasma treatment effects over varying implantation periods.

## 1. Introduction

Dental implants have been developed for more than half a century, and they have established themselves as an excellent treatment to replace lost teeth. Compared with conventional fixed and removable dentures, patients can achieve more comfortable, functional, and esthetic results by using dental implants [1,2]. Osseointegration is the most essential part of the dental implant treatment process and regeneration. There is a direct, structural, functional, durable, stable, and dynamic relation between the dental implant and the bone [3]. Typically, to obtain osseointegration, it is necessary to wait several months for a healing period after implant surgical placement [4]. In rare cases, unsuccessful osseointegration can lead to implant failure during the healing period or after the loading period due to insufficient bone quality and quantity, local infection, or various systemic diseases of the patient [5,6]. To minimize side effects or failures and to improve and promote implant osseointegration, methods for improving the design of the implant or improving the surface or design of the titanium implant have been devised [7,8,9].

Although osseointegration is improved through various implant surface enhancing methods, biological degradation, that is, aging of the implant titanium surface, can occur due to inevitable carbon-based organic impurities on the implant surface [10]. Indeed, not only the accumulation of carbon-based contaminants such as polycarbons and hydrocarbons can interfere with adhesion and proliferation of proteins and cells but also cellular and bacterial-oxide layer interactions can occur at the atomic level, resulting in bacterial adhesion and hydrophobicity [11,12,13]. Similarly, several studies have reported changes in chemical elements, particularly carbon content, associated with different surface treatments applied to titanium dental implants [14,15].

To overcome hydrophobicity associated with the occurrence of biological aging of the implant surface and increase hydrophilicity of the surface, ultraviolet (UV) light irradiation, plasma treatment, and dry and salt technology of dental implants have been devised and applied clinically [16,17]. In one study, a decrease in bioactivity appeared even one month after implant manufacturing and the decrease continued during the aging period [18]. Thus, there is a need for post-surface treatment such as UV irradiation or plasma treatment. Studies have shown that surface treatment with UV light can reduce the hydrophobic layer of hydrocarbons and carbonaceous species and increase the hydrophilicity, cell adhesion, and proliferation of implant surfaces made of titanium [19,20]. Irradiation with shortwave light can break molecular bonds and increase surface polarity, thereby increasing the reactivity of the treated surface [21]. UV-C treatment can also increase the volume of cortical-like bone tissue in the coronal region of titanium implants without deterioration of bone mineral density in rabbit femur diaphysis [22]. Plasma treatment of the implant is similar to the effect of UV light, where the surface is energized, electrons are emitted, and reactive particles are created. Increased surface reactivity by argon plasma can induce surface hydrophilization and increase cell adhesion and viability of osteoblasts without changing physical surface properties [23]. Surface treatment of implants using plasma has been studied using atmospheric pressure plasma, which discharges by injecting additional gas such as Ar or O_2_ [23].

The aim of this study was to investigate the effect of implant surface treatment with vacuum plasma (VP) on osseointegration by treating the SLA implant surface using VP without requiring additional gas. The hypothesis was that VP might enhance implant stability via implant stability quotient (ISQ) and histological osseointegration and regeneration measurements of bone-to-implant contact (BIC), bone area ratio (BA), and bone volume in rabbit experiments.

## 2. Materials and Methods 

### 2.1. Animals and Ethical Approval

This study was approved by the Institutional Animal Care and Use Committee of Yeouido St. Mary’s Hospital (YEO-2021-006FA). All surgical interventions and pre-surgical/post-surgical animal care were performed in accordance with the Laboratory Animals Welfare Act, the Guide for the Care and Use of Laboratory Animals, and the Guidelines and Policies for Rodent Survival Surgery provided by the IACUC (Institutional Animal Care and Use Committee) School of Medicine, the Catholic University of Korea. All experiments were performed in accordance with the guidelines provided by the Animal Ethical and Welfare Committee. 

Six male New Zealand white rabbits weighing about 3.5–4.0 kg were used in this study. Each rabbit was individually housed in a cage throughout the experimental period and given a standard laboratory chow. A 12 h/12 h alternating dark and light cycle was provided. There was an acclimatizing period of one week before the operation. During the entire experimental period, a veteran veterinarian provided daily ethical care to ensure the well-being of the animals.

### 2.2. Plasma Treatments

In the experimental group, the surface of each implant was treated with ACTILINK™ mini (Plasmapp Co., Ltd., Daejeon, Republic of Korea) for five minutes before surgery. After the implant was inserted into the slot of ACTILINK™ mini, it was VP-treated for 60 s in a vacuum formed by pumping for 10 s. This device features a mechanism that creates a vacuum within the package, maintaining a pressure of approximately 5 Torr. It employs a dielectric barrier discharge (DBD) configuration for plasma generation, utilizing the polyethylene packaging as the dielectric barrier and the metal body of the device as the powered electrode. The implant is grounded through a metal pumping outlet connected to the ground, enabling uniform plasma treatment across the implant surface. This approach enhances the properties of the implant surface without compromising the sterile environment [24].

### 2.3. Surgical Procedures

All surgical procedures were performed under sterile surgical conditions and sterilization protocols. All operations were performed under general anesthesia. General anesthesia was induced by intramuscular injection of a mixture of tiletamine/zolazepam (Zoletil 0.6 mL/kg, Virbac, Carros, France) and xylazine (Rompun 0.3 mL/kg, Bayer, Leverkusen, Germany).

Surgical sites were shaved and washed with an iodine solution. After 2% lidocaine solution containing 1:100,000 epinephrine was injected into the surgical site, skin incision was performed. The distal aspect of femur was exposed after dissection of muscle and periosteum. Two commercial Straumann^®^ bone level tapered (BLT) implants with SLA^®^ surface (3.3 mm in diameter, 8 mm in length, Straumann, Basel, Switzerland) were inserted into each femur using standard surgical procedures, as per the manufacturer’s instructions, under constant irrigation with sterile saline. Experimental group implants were placed in the proximal area of the femur, whereas the control group implants were placed in the distal area of the femur. There was a 1 cm distance between the centers of the implants. A total of 18 implants (10 for the experimental group and 8 for the control group) were inserted into the femurs of the rabbits. 

The surgical site was closed in layers with resorbable material (3-0 Vicryl, Ethicon, Raritan, NJ, USA). After surgery, gentamycin (50 mL, Samu Median, Seoul, Republic of Korea) and ketoprofen (20 mL, Unionbet, Seoul, Republic of Korea) were injected intramuscularly to control infection and post-operative pain for 3 days. All rabbits were sacrificed at four weeks after implantation in accordance with IACUC guidelines. The femur containing the implant was removed en bloc for radiographic and histologic analyses.

### 2.4. Implant Stability Quotient (ISQ) Measurement 

To evaluate the level of stability and osseointegration in dental implants, ISQ was measured immediately after surgery and at the time of sacrifice, four weeks after surgery. The ISQ was evaluated using the Osstell ISQ scale (Integration Diagnostics AB, Gothenburg, Sweden). Six ISQ measurements were taken for each specimen, with three measurements performed at the buccal/lingual (BL) orientation and three at the mesio/distal (MD) orientation, separated by a 90° angle. The average ISQ value was then computed from these six measurements. In general, ISQ > 70, ISQ = 60–69, and ISQ < 60 indicated high, medium, and low stability, respectively. 

### 2.5. Micro-CT Analysis 

Specimens were measured after they were fixed on a micro tomography machine (SkyScan1173; Bruker-CT, Kartuizersweg 3B, Kontich, Belgium). The area to be measured was centered. The software used for the measurement was SkyScan1173 control software (Ver 1.6, Bruker-CT). Measurement conditions were tube voltage, 130 kVp; tube current, 60 μA; filter, 1 mm aluminum; exposure time, 500 ms, pixels, 2240 × 2240; and pixel size, 12.14 μm. Rotation angles of 0.3 and 180 degrees were used. A total of 800 high-resolution images were obtained. Each specimen was scanned for a total 60 min. Resulting two-dimensional images were used to reconstruct axial cross-sections. For cross-section reconstruction, an image of 2240 × 2240 pixels was acquired using Nrecon (Ver 1.7. 0.4, Bruker-CT). Axes of cross-section images were aligned using Dataviewer (Ver. 1.5.1.2, Bruker-CT). As an analysis program, Ct Analyzer (Ver. 1.14.4.1, Bruker-CT) was used to separate and analyze bone tissues around the implant from each image. In the context of micro-CT analysis for dental implant research, “percent bone volume” quantifies the proportion of new bone formation within a defined region of interest (ROI) surrounding the implant [25]. This measurement involves a multi-step process. First, high-resolution micro-CT scanning of the implant site is performed to acquire detailed images. These images are then subjected to segmentation techniques to differentiate bone tissue from other structures. Finally, the bone volume within the defined ROI is calculated, typically expressed as a percentage of the total volume. This method provides a quantitative assessment of osseointegration and bone regeneration around dental implants, offering valuable insights into implant success and the efficacy of various surface treatments or biomaterials.

### 2.6. Histologic Analysis 

Each tissue specimen was fixed in 10% neutral buffered formalin (NBF) (MD POS, Seongnam-si, Republic of Korea) solution for 1–2 weeks. The tissue specimen was then dehydrated with increasing ethanol concentration. The dehydrated tissue sample was penetrated by increasing the resin ratio with a mixture of ethanol and Technovit 7200 (Heraeus KULZER, Hanau, Germany). After penetration was complete, the tissue was fixed in a mold and put into a UV embedding system (KULZER EXAKT 520, Germany) to harden the resin for one day. After hardening, the section was cut using an EXAKT diamond cutting system (EXAKT 300 CP, Germany) and attached to slides using Technovit 7210 (Heraeus KULZER, Hanau, Germany) and an adhesive press system. After applying Technovit 7200 to the surface, the section was re-penetrated with an infiltration system (KULZER EXAKT 530, Germany). The thickness of the final tissue section was ground to 50 ± 5 µm using an EXAKT grinding system (KULZER EXAKT 400CS, Germany). This tissue specimen was sealed and finished after staining. H&E staining (HE), Goldner’s trichrome staining (GT), and von Kossa staining (Vk) were performed with each commercial kit (StatLab, McKinney, TX, USA) according to the manufacturers’ instructions. 

The final-produced incline tissue slides were obtained with various digital images using a Pannoramic 250 Flash III of 3D Histech (No. 3 Öv Street, Budapest, Hungary). After obtaining digital images, BIC and BA were measured using Image Pro plus^®^ (Media Cybernetics, Inc., Rockville, MD, USA). Two primary parameters were evaluated: bone-to-implant contact (BIC) and bone area (BA). Bone-to-implant contact (BIC) was calculated to assess the degree of osseointegration. This measurement involved determining the percentage of the implant surface in direct contact with bone tissue. The total length of the implant surface (TL) was first measured, followed by the cumulative length of bone tissue in direct contact with the implant surface (BL). BIC was then calculated using the formula BIC (%) = (BL/TL) × 100. This percentage represents the proportion of the implant surface that has achieved direct bone contact, serving as a quantitative measure of osseointegration. Bone area (BA) was evaluated to quantify the amount of bone tissue present in the peri-implant region. For this analysis, a region of interest (ROI) was defined around the implant. Within this ROI, three measurements were taken: the total area of the defined region, the area occupied by the implant itself, and the area occupied by bone tissue. The total analyzable area was calculated by subtracting the implant area from the total ROI area. BA was then computed using the formula BA (%) = (bone tissue area/total analyzable area) × 100. This percentage represents the proportion of the peri-implant region occupied by bone tissue, providing insight into the quantity and quality of bone formation around the implant.

### 2.7. Statistical Analysis

All statistical analyses were performed using SAS software version 9.4 (SAS Institute, Cary, NC, USA). The difference between the experimental group and the control group was compared through an independent *t*-test or Wilcoxon’s rank sum test by testing whether data were normally distributed. Mean, standard deviation, median, minimum, and maximum values are presented. In addition, paired *t*-test or Wilcoxon’s signed rank test was performed for comparison between ISQ difference (at operation and osseointegration) within each group. All statistical significance levels were set at *p* < 0.05. 

## 3. Results

### 3.1. Results of Micro-CT Analysis

As a result of comparing percent bone volume through micro-CT analysis, the experimental group had a percent bone volume of 61.47 ± 8.14, which was higher than that (52.50 ± 18.78) of the control group (Table 1). However, the difference between the two groups was not statistically significant (*p* > 0.05).

### 3.2. Results of Histologic Analysis

**Figure 1:**
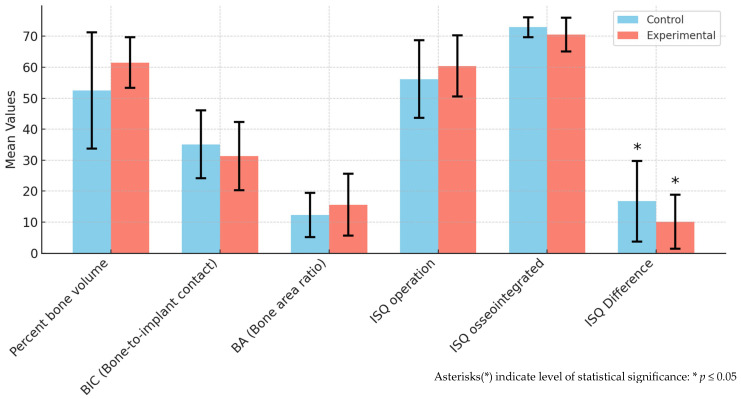
Results of micro-CT, histologic analysis, and implant stability quotient (ISQ) comparison.

**Figure 2:**
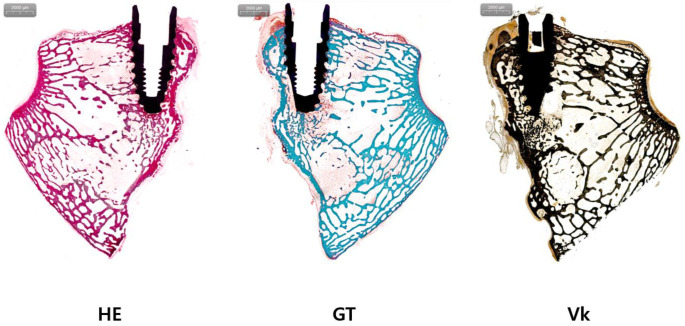
Histologic analysis of the control group. H&E staining (HE), Goldner’s trichrome staining (GT), and von Kossa staining (Vk) were performed for the control group.

**Figure 3:**
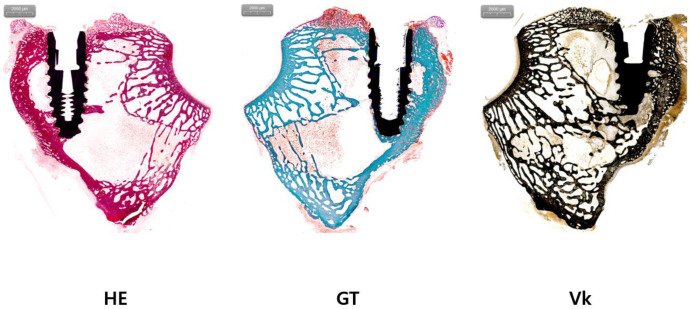
Histologic analysis of the experimental group. H&E staining (HE), Goldner’s trichrome staining (GT), and von Kossa staining (Vk) were performed for the experimental group.

BIC and BA were compared through histological analysis. In the experimental group, BIC was 31.30 ± 11.06 and BA was 15.61 ± 9.98. In the control group, BIC was 35.09 ± 10.92 and BA was 12.31 ± 7.17 (Table 1, Figure 1, Figure 2 and Figure 3). The experimental group showed a slightly higher BA, and the control group showed a slightly higher BIC. However, differences between the two groups were not statistically significant (both *p* > 0.05).

### 3.3. Results of ISQ Analysis

The ISQ value increased significantly from 58.51 ± 11.01 at the time of implantation to 71.56 ± 4.63 at the time of sacrifice (Table 1 and Figure 1). Within each group, the changes in ISQ values before and after the experiment showed statistically significant differences (*p* ≤ 0.05). However, there was no significant difference observed between the experimental and control groups (*p* > 0.05).

## 4. Discussion 

The percent bone volume was 52.50 ± 18.78 for the control group and 61.47 ± 8.14 for the experimental group, indicating a slight, but not statistically significant, increase in the experimental group. The BIC was 35.09 ± 10.92 in the control group and 31.30 ± 11.06 in the experimental group, again showing a non-significant difference. Similarly, BA was 12.31 ± 7.17 in the control group and 15.61 ± 9.98 in the experimental group, with no significant difference. The ISQ value increased significantly from 58.51 ± 11.01 at implantation to 71.56 ± 4.63 at the time of sacrifice, with no significant difference between the groups. In summary, while the experimental group showed somewhat higher BA and percent bone volume, these differences were not statistically significant, indicating no significant difference in osseointegration between the groups.

Plasma treatment of the implant surface can have a positive effect on osseointegration by increasing hydrophilicity of the implant. The hydrophilicity of the implant surface is one important factor that can promote osseointegration. A hydrophilic surface of the implant has a higher affinity for protein than a hydrophobic surface, allowing the protein to maintain its conformation and function. Stable adsorption of proteins to the implant can affect the ability of cells to adhere and migrate to the implant surface. In addition, high hydrophilicity can promote differentiation and maturation of osteoblasts, thereby promoting osseointegration [9]. Another in vitro study proposed a vacuum plasma device for the surface treatment of dental implants, demonstrating significant improvements in osseointegration efficiency, including enhanced protein adsorption, cell adhesion, and proliferation, without damaging the implant’s calcium coating [24].

Plasma treatment can increase hydrophilicity of the implant by increasing the generation of hydroxyl groups on the implant surface. When the surface of the implant made of TiO_2_ was treated with oxygen plasma for more than 150 s, the contact angle showed hydrophilicity of 0 degrees. After treatment for 300 s, the hydroxyl group structure was increased by 35.8% [26]. Plasma treatment on the implant surface enhances hydrophilicity by reducing carbon residues and exposing the inherent implant material. XPS analysis of SLA disk samples treated with non-thermal atmospheric pressure plasma showed a reduction in carbon from 43.59% to 20.72% and an increase in Ti from 12.43% to 18.44%. In vitro experiments demonstrated that plasma-treated samples had increased protein adsorption and spindle-shaped cells with expanded actin filaments attached to the surface [27]. In addition, when an oxygen plasma treated-hydrophilic sample was implanted into the tibia of a rabbit, greater force was required during a pull-out test [28]. In a recent air atmospheric pressure plasma jet (AAPPJ) in vitro study, results indicated that AAPPJ treatment might lead to a 20% increase in early attachment and proliferation of HGF for establishing early peri-implant soft tissue seals on titanium dental implant with possible favorable effects of osseointegration of dental implant [29]. Implants plasma-treated with Ar and O_2_ gas showed a decrease in carbon content on the surface but an increase in hydrophilicity. As a result of placing plasma-treated samples in beagles, more newly generated bone tissues were observed than samples without a plasma treatment [30]. 

In this study, the difference between the experimental group and the control group was not significantly different. The application period of the placed implant could be one reason. In the case of a hydrophilic SLA implant with a hydroxylated oxide surface due to a change in chemical structure, reactions with ions, amino acid, and proteins in the tissue fluid might be more active. As a result, BIC values of implants with hydrophilicity were significantly enhanced only in the initial stage of bone regeneration as the healing period decreased. The difference was insignificant in subsequent periods [8]. Similarly, the SLA implant treated by plasma under an atmosphere of oxygen with hydrophilicity due to the produced OH group showed a significant difference in BIC values only in the early bone healing stage. However, differences were insignificant in subsequent periods [31]. It means that the effect of plasma treatment on the implant surface has a greater effect in the early stage of osseointegration. For this reason, it can be considered that the difference in the effect of the VP-treated implant on bone formation might be small at four weeks after implantation because the implant treated with VP could promote initial bone healing. Therefore, further studies are needed to confirm the effect of plasma treatment on osseointegration according to the implantation period.

The positive effect of plasma treatment on the implant surface is observed not only immediately after the treatment, but also persists over time [32]. When the implant was treated with Ar plasma, amounts of carbon present on the implant surface, before plasma treatment (control), immediately after plasma treatment, and after 30 days after plasma treatment were 32.91%, 15.25%, and 24.6%, respectively [32]. Although the amount of carbon on the implant surface was increased after 30 days of plasma treatment, it was maintained lower than that of the control. In addition, when a sample at 30 days after plasma treatment was applied to a beagle, the BIC was similar. However, bone area fraction occupancy (BAFO) and removal torque values were higher than those of the control. The plasma treatment method used in previous studies was performed at atmospheric pressure by inserting additional gases such as Ar or O_2_. On the other hand, the plasma treatment used in this study did not require additional gas and was discharged under low pressure conditions such as vacuum. This VP treatment is more convenient than atmospheric pressure plasma as it does not require additional gas management or maintenance. In addition, the VP is known to have higher electron energy than atmospheric pressure plasma treatment [33]. This high energy is expected to be able to remove more carbon by reacting more with the carbon present on the implant surface. Results of a recent study revealed that UV light treatment or atmospheric pressure plasma treatment could lead to rather high BIC and BAFO, with a positive effect on the early stage of osseointegration using zirconia implants in other pig animal experiments [34]. However, even in the mentioned study, the control group and the experimental group did not show a significant difference, which was thought to be influenced by the implant material related to the aging problem of the implant surface [34]. In a study comparing the enhancement of implant surface properties and hydrophilicity due to physical changes—whether titanium or ceramic—various physical methods of altering surface roughness did not significantly improve surface properties such as hydrophilicity [35,36]. After plasma treatment using a commercial Straumann SLA implant, carbon content decreased, while protein adhesion and cell adhesion increased [37]. It is expected that VP treatment will be more clinically helpful in improving the implant surface and improving osseointegration. In another study, cold atmospheric plasma has also been shown to be effective in decontaminating titanium implant surfaces [38]. In addition to improving the properties of the implant surface itself, plasma treatment can also enhance implant success by reducing bacterial presence as an additional effect.

A primary limitation of this study is the relatively small sample size, chosen to minimize the use of animals in accordance with ethical guidelines, which may have reduced the statistical power to detect significant differences between groups. Additionally, the absence of weekly ISQ measurements—due to concerns about animal welfare associated with frequent anesthesia and handling—limited our ability to monitor dynamic changes in implant stability over time. Furthermore, the lack of direct measurements of implant surface hydrophilicity prevents us from conclusively linking the VP treatment to changes in surface wettability that may influence osseointegration. Despite these limitations, our findings highlight the potential for implant surface aging—particularly in titanium or titanium alloy fixtures during routine procedures—and underscore the importance for dentists and biomedical engineers to recognize this possibility and develop proactive strategies to address it. VP treatment emerges as an effective method to clean and reactivate aged implant surfaces, restoring their biocompatibility and enhancing osseointegration, while offering practical advantages in clinical settings such as ease of application and reliable outcomes. Addressing the limitations of this study in future research—with larger sample sizes, more frequent non-invasive measurements, and comprehensive surface analyses—will be crucial to validate and expand upon our findings.

## 5. Conclusions

The purpose of this study was to evaluate the effect of VP treatment on the osseointegration and regeneration of dental implants in a rabbit model. Our findings indicated that bone volume, BIC, BA, and ISQ values were generally higher in the plasma-treated experimental group compared with the control group after four weeks of healing. However, these differences were not statistically significant.

All implants, irrespective of the surface treatment, demonstrated successful osseointegration and regeneration. This suggests that, while VP treatment may have the potential to enhance certain aspects of implant integration, the effects were not conclusive within the limitations of this study, which include a relatively small sample size and the absence of direct measurements of surface hydrophilicity. Future studies with larger sample sizes, extended observation periods, and comprehensive surface analyses—including direct measurements of hydrophilicity—are necessary to further investigate the impact of VP treatment on implant osseointegration and longevity.

## Figures and Tables

**Table 1:** Results of micro-CT, histologic analysis, and implant stability quotient (ISQ) comparison.

		Control	Experimental	Total
Percent bone volume in micro-CT	N	8	10	18
	Mean (SD)	52.50 (18.78)	61.47 (8.14)	57.48 (14.19)
	Median	52.35	64.25	55.47
	Min, Max	17.78, 83.51	50.80, 71.51	17.78, 83.51
	Between *p*-value	0.2393 (T)		
BIC (bone-to-implant contact)	N	8	10	18
	Mean (SD)	35.09 (10.92)	31.30 (11.06)	32.99 (10.85)
	Median	35.19	32.21	32.81
	Min, Max	16.46, 54.40	16.60, 45.78	16.46, 54.40
	Between *p*-value	0.4781 (T)		
BA (bone area ratio)	N	8	10	18
	Mean (SD)	12.31 (7.17)	15.61 (9.98)	14.15 (8.76)
	Median	12.56	16.2	14.27
	Min, Max	2.84, 24.78	1.26, 31.79	1.26, 31.79
	Between *p*-value	0.4436(T)		
ISQ at operation (POD#0)	N	8	10	18
	Mean (SD)	56.15 (12.51)	60.40 (9.91)	58.51 (11.01)
	Median	56.25	59.5	58.92
	Min, Max	35.00, 74.00	45.00, 74.50	35.00, 74.50
	Between *p*-value	0.4318 (T)		
ISQ at osseointegration (POD#1M)	N	8	10	18
	Mean (SD)	72.88 (3.18)	70.50 (5.46)	71.56 (4.63)
	Median	72.83	69.75	71.33
	Min, Max	68.00, 77.00	61.83, 78.00	61.83, 78.00
	Between *p*-value	0.2928 (T)		
ISQ difference (0–1 M)	N	8	10	18
	Mean (SD)	16.73 (12.98)	10.10 (8.72)	13.05 (11.00)
	Median	19.25	11.83	12.92
	Min, Max	−3.00, 33.00	−8.67, 23.50	−8.67, 33.00
	Within *p*-value	0.0082 * (P)	0.0052 *(P)	0.0001 * (P)
	Between *p*-value	0.2137 (T)		

Within *p*-value: paired *t*-test (P) or Wilcoxon signed rank test (S). Between *p*-value: independent *t*-test (T) or Wilcoxon rank sum test (W). Asterisks indicate level of statistical significance: * *p* ≤ 0.05.

## Data Availability

Data can only be provided or accessed from the corresponding author upon reasonable request.
